# Vaccines for the Allied Armies

**Published:** 1916-03-04

**Authors:** 


### VACCINES FOR THE ALLIED ARMIES.

### The Inoculation Department at St. Mary's Hospital.

The value of preventive inoculation is one of the great lessons the present war has taught us. At various times in The Hospital, notably in the issue of January 29 (p. 358), it has been shown how, as a direct consequence of inoculating the troops of all the belligerent countries, the incidence of typhoid has been almost a negligible factor when compared with the ravages caused by this disease in recent campaigns. It is not, however, the purpose of this article to enlarge upon the striking rarity of typhoid fever among inoculated men and the consequent economy in fighting material, but rather to consider the means—or one of the means—by which it has been possible in the time of a great crisis to increase the output of vaccines so enormously as to enable millions of men to receive protective treatment.

Although when the war broke out the machinery existing in England for making vaccines on such a colossal scale as to meet the needs of the new armies was wholly inadequate, it was in such capable hands that it was soon extended to fulfil all the demands that were placed upon it. It is true that in the early days of the campaign the resources of the United States had to be drawn upon, but for all practical purposes this country is now not only self-dependent in this respect, but has long been furnishing antitoxins to the armies of our Allies. The accomplishment of this task has been due to the initiative and energy of the inoculation department at St. Mary's Hospital, without which our armies would have been in a sad plight. Indeed, it is not too much to say that had not this department been equal to the occasion it would hardly have been possible to inoculate more than a proportion of our soldiers, and typhoid epidemics would probably have been a common experience of the campaign, since the natural conditions under which our army has been fighting for so long are just such as are favourable to typhoid.

### A Hospital Factory.

Before the outbreak of war it was already part of the policy of this department—which is self-supporting—to prepare vaccines on a commercial scale; but the production in those days was small compared with the output at the present time; and what was before the war an extensive and well-equipped laboratory in which the preparation of antitoxins for distribution was subsidiary to the general work of bacteriological investigations, is to-day more like a large manufacturing house in which all the processes of the great business of making, bottling, labelling, packing, and dispatching large consignments of goods are carried on.

There are, of course, commercial laboratories in other countries, notably in America and Germany, where the same sort of thing is going on; but where else shall we find a hospital having as one of its departments a factory which, besides supplying the home demand, traffics in its strange wares with the great countries of Europe, and supplies millions of men with prophylactics against disease? During the first year of the war the department prepared and dispatched to their proper destinations nearly five million doses of vaccines. Anti-typhoid vaccine formed by far the largest proportion of this bulk, but cholera and antisepsis vaccines were also supplied; in addition to our own armies the French, Russian, Belgian, and Serbian troops received large quantities of vaccines from this source. The output during the first six months of the second year of the war has been on a still larger scale; but, despite the difficulties of working with a depleted staff and other obstacles, such as the great dearth of bottles and ampoules, the work is going on at the same rapid pace.

### The Preparation of "T.A.B."

At the present time the preparation of Paratyphoid A and Paratyphoid B vaccines is engaging special attention, and a mixed vaccine consisting of typhoid and these two vaccines—known as "T.A.B."—is being produced in considerable quantity. At the time of the writer's visit this mixed vaccine was in course of preparation, and he was enabled to witness several of the processes through which it has to pass.

Of all the processes through which an antitoxin has to pass from the catching of the microbe to the dispatching of the packed cases of the finished product, the most difficult is sometimes the first. Take the case, for instance, of cholera vaccine. In order to obtain the strains a member of the staff went to Galicia, and at Przemsyl, which was then in Russian occupation, procured the required bacteria from cholera patients. More recently half a million cc. of cholera vaccine—for which strains were sent from the fighting lines in Poland—have been supplied to the Russian Army. Cholera vaccine has also been supplied to the Serbian Army. The paratyphoid strains were isolated from British soldiers back from the Front, while the bacteria from the preparations of the anti-gangrene were isolated by Sir Almroth Wright in France.

If one's visit to this department is happily timed, it is possible to witness the process of identifying the strain and of ascertaining if it is true to type, the growing of it in bulk in the particular way that suits the particular microbe, the making of the emulsion, the counting and the killing of the microbes, the dilution to the proper strength, as well as the bottling, labelling, and packing of the finished products. All this work has to be done by a staff that is fluctuating, but perforce always small in consequence of the other demands of the war. Indeed, at the most all the department can count upon is a *personnel* of fourteen, of whom five are qualified and nine unqualified. It is, therefore, obvious that on occasion the staff of what may not inappropriately be described as the manufacturing side of the department has to work all hours of the day and night.

